# IgG Avidity Test in Congenital Toxoplasmosis Diagnoses in Newborns

**DOI:** 10.3390/pathogens6020026

**Published:** 2017-06-18

**Authors:** Zulmirene Cardoso Fonseca, Isolina Maria Xavier Rodrigues, Natália Cruz e Melo, Juliana Boaventura Avelar, Ana Maria Castro, Mariza Martins Avelino

**Affiliations:** 1 Department of Gynecology and Obstetrics, Faculty of Medicine, Federal University of Goias (UFG), Goiania GO 74045-155, Brazil; mariza.avelino@gmail.com; 2 Laboratory of Immunology of Clinical Hospital of UFG, Goiania GO 74605-020, Brazil; isolinaxavierrodrigues@gmail.com; 3 Department of Gynecology, Faculty of Medicine, Federal University of São Paulo, São Paulo SP 04039-032, Brazil; melo.natalia@unifesp.br; 4 Department of Microbiology, Immunology, Parasitology and Pathology, Institute of Tropical Pathology and Public Health, UFG, Goiania GO 74605-450, Brazil; jubavelar@hotmail.com (J.B.A.); amaria@ufg.br (A.M.C.)

**Keywords:** congenital toxoplasmosis, IgG avidity, diagnoses, newborns, puerperae

## Abstract

The goal of this study was to investigate the importance of IgG avidity testing in newborns (NBs) diagnosed with early congenital toxoplasmosis. We collected samples from 88 puerperae infected by *Toxoplasma gondii* (*T. gondii*) and their NBs (48 acutely-infected puerperae (AIP) and 40 chronically-infected puerperae (CIP)), from two public maternity hospitals in Goiania city, Goias, Brazil, from 2010 to 2015. Specific anti-*T. gondii* IgM and IgG serum levels and IgG avidity tests were evaluated using chemiluminescence. Congenital toxoplasmosis was observed in 66.66% (*n* = 32) of NBs with AIP, 94.1% presenting low avidity (LA) and 51.61% presenting high avidity (HA) test results. The IgG and IgM levels of NBs with LA and their puerperae were higher in comparison with HA NBs and puerperae (*p* = 0.0001). The avidity tests showed 100% specificity and 50% sensitivity (*p* = 0.0001). NBs with LA had a 15-fold increased risk of developing congenital toxoplasmosis in comparison with HA NBs. The IgG avidity test could be used to assist in early congenital toxoplasmosis diagnoses in NBs and LA, identifying a greater probability of vertical transmission.

## 1. Introduction

*Toxoplasma gondii* (*T. gondii*) is an obligate intracellular parasitic protozoan that causes toxoplasmosis [1,2]. This parasite can be acquired through contaminated food ingestion, blood transfusion, organ transplantation, and by vertical transmission [3,4,5,6,7]. The infection caused by *T. gondii* is usually asymptomatic but its most severe manifestation is congenital toxoplasmosis [5,8,9].

Congenital toxoplasmosis occurs in different regions of the world with an incidence of 1–14 cases per 10,000 pregnancies [10,11,12]. In Brazil, it is estimated that for every 10,000 live births, 5–23 newborns are infected with *T. gondii* [13]. In addition, studies have reported high seroprevalence of anti-IgG *T. gondii* in Brazilian pregnant women previously exposed to the parasite [14,15,16,17,18], which increases the risk of infection of the fetus in pregnancy.

Newborns affected by congenital toxoplasmosis may present a broad spectrum of clinical manifestations, ranging from the normal phenotype to pathological changes, such as the classic triad (hydrocephaly, chorioretinitis, and intracranial calcifications) [19,20], abortion, fetal death, low birth weight, blindness, hearing loss, severe cognitive deficiencies, and hydropsy [19,20,21].

To prevent the serious manifestations of *Toxoplasma gondii* in newborns, it is necessary to monitor pregnant women during prenatal and postnatal care, and to follow up with newborns of pregnant women at risk. However, there are limitations. Toxoplasmosis during pregnancy represents a clinical challenge because it is subclinical in most infected women [22,23]. The serological evaluation to determine the moment in which the *T. gondii* infection was acquired, and the corresponding gestational phase, is relevant for intervention and treatment since the acute phase of the infection can lead to clinical manifestations in pregnant women and congenital toxoplasmosis [5,24,25]. The presence of specific IgG-class antibodies in pregnant women suggests previous infection, and positive IgM levels—in combination with positive results for IgG—usually suggest recent infection [19,26]. However, the identification of a recent infection can be complicated because IgM antibodies can be detected after several months or years of a primary infection [27,28]. 

The diagnosis of infected newborns is difficult, because the presence of specific IgG in newborns does not mean disease, since the maternal IgG crosses the placental barrier [27,29]. The specific IgM is not usually present in newborns, since the high maternal antibody titers of IgG passing through the placenta inhibit fetal IgM formation [22,27], which induces a false negative laboratory result. Therefore, it is necessary to perform various diagnostic tests to identify asymptomatic- or oligosymptomatic-infected newborns. The purpose of these tests are to identify: (1) the presence of sequelae lesions resulting from congenital infection, such as ocular alterations and intracranial calcifications, through fundus and ultrasound exams [27,30,31]; (2) the etiological DNA agent by PCR (in blood or in cerebrospinal fluid) [32,33,34]; and (3) the presence of specific IgG antibodies (to be compared with the levels of antibodies of the mother) and the presence of IgM and IgA in the blood of the newborn [22,27]. These tests can be of assistance in the identification of congenital toxoplasmosis allowing early treatment of infected newborns, thereby reducing the severe manifestations of toxoplasmosis [35,36]. However, it is still necessary to search for a new method of congenital toxoplasmosis diagnosis in newborns, which might be economically feasible, with easy methodology allowing for the clinical management of infected newborns.

The IgG avidity test, first described by Hedman et al. [37], evaluates the binding avidity of IgG antibodies against *T. gondii*, separating the low-avidity (LA) antibodies produced in the initial phase of the infection from the high-avidity (HA) antibodies produced in chronic infection [37,38,39,40]. Studies have proposed the IgG avidity test as an important method to identify the acute phase of toxoplasmosis in pregnant women, showing 100% sensitivity and 92.7% specificity [39,41], but little is known about the value of this test in newborns. Following this approach, the study of Buffollano et al. [42] evaluated the association of IgG avidity testing in congenital toxoplasmosis detection in newborns. The obtained data showed that most infected newborns presented LA values and these reflected the maternal values. To investigate this association, the objective of the present study was to verify the importance of the IgG avidity test for the diagnosis of congenital toxoplasmosis in newborns.

## 2. Results

Based on the results of IgG avidity testing, the NBs were divided into three groups: (a) two groups were composed of newborns (NBs) from acutely-infected puerperae: (a1) NB with low avidity of IgG (*n* = 17) and (a2) NB with high avidity of IgG (*n* = 31), and (b) a group of NB from chronically-infected puerperae (*n* = 40). The correspondence of IgG avidity tests in NBs and the puerperae was 88% (15/17) in low avidity of IgG (LA) and 96.7% (30/31) in high avidity of IgG (HA), and the control group presented 100% (40/40) correspondence in pregnant women with high avidity and their newborns, with *p* = 0.0001 (Table 1).

When we analyzed the correlation of IgG, IgM, and avidity in the RNs and their puerperae we verified a directly proportional correlation between IgG levels (*r* = 0.809) and IgM (*r* = 0.835) with *p* < 0.001. However, between the values of avidity and serum levels of IgM in RNs and their puerperae, an inversely proportional correlation was observed (*r* = –0568; *p* < 0.001) (Table 2). Therefore, our results indicate that when IgG levels are elevated, the IgM levels are also increased. The reverse occurs between levels of IgM and avidity; in other words, when the IgM are increased, avidity is reduced.

**Figure 1:**
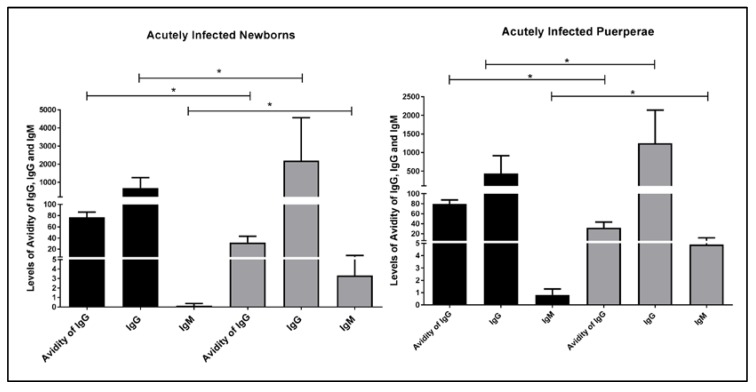
Blood levels of IgG, IgM, and anti-*Toxoplasma gondii*-specific IgG avidity test values in newborns and their puerperae infected by *Toxoplasma gondii*, Goiania, Goias, Brazil, 2016. IgG avidity test values, IgM, and IgG of infected NBs (**left**) and their purperea (**right**) with IgG high avidity are displayed in black (*n* = 16), whereas low avidity (*n* = 16) are displayed in gray. The levels of IgG, IgM, and IgG avidity were measured using the chemiluminescent technique. Test: unpaired Student’s *t*-test * represents the existence of significant difference (*p* < 0.005).

The congenital toxoplasmosis was observed on 66.66% (32/48) of NBs of AIP. In the group of NBs with LA, it was found that 94.11% (16/17) of NBs were infected, while 51.61% (16/31) of NBs with HA were infected. Levels of IgG, IgM, and IgG avidity were verified in NB and AIP infected with *T. gondii* (Figure 1). We observed higher levels of IgM and IgG in the NBs with LA and their puerperae in comparison with the NBs with HA and their puerperae (*p* = 0.0001). Interestingly, it was verified that NBs and their puerpera showed similar expression profiles of IgG, IgM, and IgG.

The presence of clinical signs was observed in 43.8% (14/32) of NBs infected. In relation to IgG avidity test values and the presence of symptomatology, it was verified that 56.2% (9/16) of NBs with LA and 31.3% (5/16) of NB with HA showed clinical manifestations compatible with congenital infection (Table 3). The NBs in the present study generally showed more than one clinical sign of the disease. 

Among clinical manifestations found in NBs with LA, severe central nervous system (CNS) impairment (5/9), such as hydrocephaly (4/9) or microcephaly (1/9), were observed. In addition, verified blindness (2/9) and ocular defects associated with cerebral involvement, characterizing a neuro-optic form (2/9) were also observed. In the case of NBs with IgG HA, cerebral calcifications (4/5) and generalized lymph node form (1/5) were observed. Thus, we can observe that there is a significant difference in relation to the presence of clinical signs of congenital toxoplasmosis between the infected NBs with low and high avidity (*p* = 0.0006), as observed in Table 3. 

**Figure 2:**
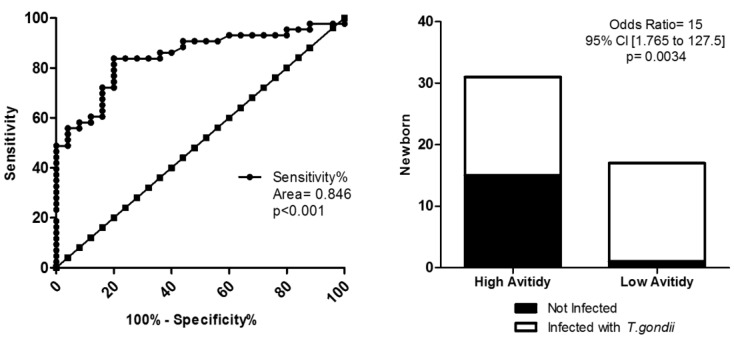
Avidity test in diagnostic of congenital toxoplasmosis of newborns based in low avidity. (**A**) ROC curve for the <48% cutoff, showing 100% sensitivity and 50.0% specificity, *p* = 0.001. (**B**) Odds ratio presented with a 15-fold increase in the risk of developing congenital toxoplasmosis for newborns with low avidity, *p* = 0.0034. ROC curve, odds ratio, and Fisher’s test were utilized, considering *p* < 0.005 as a statistically significant difference.

The avidity test values in diagnosis and congenital toxoplasmosis prediction was evaluated in NBs (Figure 2). Using the cut-off of IgG avidity of <48%, observed specificity of 100%, and sensitivity of approximately 50% (area = 0.846; *p* = 0.001). In addition, the odds ratio revealed that NBs with LA presented a 15-fold increase of risk to develop congenital toxoplasmosis than NBs with HA (*p* = 0.034).

## 3. Discussion

Some studies have demonstrated the avidity of IgG as an important method to identify the acute phase of toxoplasmosis, with 100% sensitivity and a specificity of 92.7% [39,41]. The avidity test value in pregnancy is being established [18,40,44], but IgG avidity detection in NBs is still not elucidated and the few avidity studies on NBs reported LA in neonates infected with congenital toxoplasmosis [18,42].

Data from the literature have already revealed that patients with chronic *T. gondii* infection have high avidity and acute infection patients generally present with low avidity [40,44]. The high IgG avidity in pregnant women before the first trimester of pregnancy can indicate infection later, which makes this test useful in the beginning of pregnancy. However, this finding does not exclude the possibility of fetal involvement occurring during the pregnancy [15], and detection of high avidity at the end of pregnancy does not eliminate an acquired infection in the first trimester or gestational second trimester [26], although some studies have shown that high avidity in the pregnant woman indicates a probable reduction of the risk of fetal infection [19,41,44]. On the other hand, the detection of LA in puerperae generally indicate recent infection, which would increase the risk of vertically maternal toxoplasmosis [19,44], but, some studies have reported low avidity after a long period of recent infection [45,46]. Therefore, studies are necessary to understand the role of the avidity test in the diagnosis of toxoplasmosis in the pregnant woman and in the identification of infected newborns.

Table 1 shows that both acutely-infected and chronically-infected puerperae and their newborns presented the same IgG avidity profile. The correlation of IgM and IgG-specific IgG blood levels between NB and its puerperae was directly proportional, whereas IgM levels and the avidity test showed an inverse proportional relationship, that is, the increase in IgM was associated with LA. Several studies have already observed that the increase in IgM is a marker of recent infection and that BA associated with high levels of IgM can indicate acute toxoplasmosis [18,19,42,44].

Congenital toxoplasmosis was verified in 66.66% (32/48) of RNs from AIP, being that 94.11% (16/17) of NBs with LA were infected, while 51.61% (16/31) of NBs with HA were infected. In the serological analysis, the NBs infected with LA and their puerperae show an increase of specific IgM and IgG in relation to NBs infected with HA and their puerperae, with statistical significance. Interestingly, puerperae and NBs presented the same serological profile of the IgG, IgG, and IgM avidity (Figure 1 and Table 1), and the concordance of these parameters was already reported in other studies [42,47]. 

Some studies have addressed the relationship between LA values in pregnant women as a likely increase in the risk of vertically maternal toxoplasmosis [19,44]. However, other studies show that, due to immunological changes during pregnancy, or in response to antibiotic treatment [45,46], the avidity of IgG may remain low for a long period following infection [10,48]. However, few studies have investigated this relationship in newborns, and our findings corroborate the results of Buffollano et al. [42], which demonstrated the association of LA values in neonates infected with congenital toxoplasmosis, and contradict the findings of Fidal et al. [49], who did not observe congenital infection in newborns of women with low avidity.

The symptomatology of congenital toxoplasmosis was observed in 43.8% (14/32) of infected NBs. Meaning that the NBs with LA presented a greater number of symptoms compatible with congenital infection than the NBs with HA, NBs with LA presented more than one clinical sign of the disease. Among severe clinical manifestations, hydrocephalus, microcephaly, blindness, neuro-optic involvement, and generalized toxoplasmosis with neonatal death were observed. Severe forms of congenital toxoplasmosis occur more frequently in the absence of prenatal care. However, in the state of Goias, there is a prenatal program for congenital toxoplasmosis. This result suggests the existence of failures in primary and secondary measures, which may also be associated with the presence of more aggressive *T. gondii* strains in the state [15,50,51]. Hence, it is necessary to develop new strategies to establish a more effective program for congenital toxoplasmosis prevention. 

In summary, our data showed that NBs exposed to *T. gondii* with LA IgG presented increased serum levels of specific IgM and IgG, exhibited more severe congenital toxoplasmosis symptoms, and had a 15-fold greater risk of developing toxoplasmosis than NBs exposed to *T. gondii* with HA, data not previously described in the literature.

In addition, low IgG avidity testing in NBs for congenital toxoplasmosis diagnosis with cutoff < 48% presented 100% specificity and 48.30% sensitivity, with *p* = 0.001 (Figure 2). Despite the test’s low sensitivity, it still surpassed the sensitivity of specific IgM and IgA with regard to diagnosing congenital infection. These data reinforce that the avidity test could also be an important tool to assist in the clinical management of the newborns with congenital toxoplasmosis and, to our knowledge, the prognostic value of this test in congenital infection has yet not been described. 

However, the present study has limitations, RNs with high avidity were also infected with *T. gondii* and some studies detected low avidity in the chronic phase of the disease. Thus, there are still questions to be answered about the role of the avidity test in the early diagnosis of congenital toxoplasmosis in newborns, which could be elucidated in studies with a larger sample. Despite this, we caution the importance of the monitoring of NB with LA for the early detection of congenital toxoplasmosis.

The results of our study showed that the combination of specific anti-*T. gondii* IgM in NB peripheral blood, together with LA IgG in NBs, increased congenital toxoplasmosis diagnosis prediction in NBs and suggested greater severity of congenital disease. In pregnant women, data from the literature have revealed a relationship between acute toxoplasmosis with LA IgG [26,39,40]. In addition, Robert et al. [38] showed that the use of both tests (IgM and IgG avidity testing) presented 99% specificity in acute toxoplasmosis infection diagnoses. These data reinforce the importance of using avidity testing to assist in the clinical management of NBs with congenital toxoplasmosis. 

## 4. Materials and Methods

### 4.1. Type of Study and Population 

A retrospective case control study was nested in a cohort (control of vertical transmission of congenital toxoplasmosis in Goias), in the period from 2010 to 2015, in patients that attended two public maternity hospitals in Goiania, Goias, Hospital das Clinicas of the Federal University of Goias (HC/UFG) and the Maternity Our Lady of Lourdes (MNSL). In these health centers, IgM and IgG analysis specifically for *T. gondii* are performed routinely in prenatal care, patients infected during the study period were followed up until the birth of the newborn. The collected serum was maintained in a refrigerated state until the tests, which were processed in duplicate within a maximum of 24 h after collection. Forty-eight (48) acutely-infected puerperae (AIP) by *T. gondii* were identified during pregnancy, attended at HC/UFG, Reference Service for Vertical Transmission Control of Toxoplasmosis in Goias. In addition, we studied 40 chronically-infected puerperae (CIP) (IgM-negative), attended at MNSL (Reference Service of Low-Risk Pregnancies), and their respective newborns.

### 4.2. Ethical and Legal Aspects

This research was approved by the Ethics Committee in Human and Animal Medical Research of the Clinical Hospital of Federal University of Goias (CEP/HC-UFG), No. 039/2002, because the study respected and followed the precepts established in Resolution 466/12 of the National Health Council 10/10/1996. The patients signed free informed consent forms.

### 4.3. Inclusion Criteria

The Reference Service of Congenital Infection Care at the Clinical Hospital of Federal University of Goias (Control of Toxoplasmosis Vertical Transmission) is in Goiania city and performs the follow-up of acutely-infected pregnant women and their children suspected of congenital infection. To establish *T. gondii* infection in the pregnant woman, serum levels of IgM and IgG specific for *T. gondii* and avidity of IgG were measured. Pregnant women with specific IgM and IgG reagents were considered to be acutely infected, while pregnant women with specific IgG reagent and high avidity were considered to be chronically-infected. 

The NBs suspected of congenital infection (NBs of pregnant women with anti-*T. gondii*-specific IgM in pregnancy) underwent complete clinical investigation with specific anti-*T. gondii* serological tests, polymerase chain reaction (PCR), transfontanel skull ultrasonography, fundoscopy, and cerebrospinal fluid analysis. To establish a confirmatory diagnosis of congenital toxoplasmosis in follow-up NBs, the following criteria were used by a trained clinician:

1. Presence of anti-*T. gondii* IgM and/or IgA in any concentration (above the cut-off) after the fifth day of birth. These immunoglobulins can pass to a NB during labor, but they do not cross the placental barrier and their encounter after the fifth day of birth (average lifespan) is diagnosed as congenital infection;
2. High anti-*T. gondii* IgG (greater than or equal to 300 IU/mL), associated with clinical alterations compatible with congenital toxoplasmosis;
3. Cerebrospinal fluid abnormalities, such as the presence of antibodies of the IgG class (measured thought indirect immunofluorescence), the identification of *Toxoplama gondii* by PCR, and the inoculation of the CSF of the newborns suspected of congenital infection in mice;
4. Increase in IgG concentration or maintenance of elevated levels for more than three months with or without clinical infection manifestations;
5. IgG presence in greater concentration than that from the mother, in at least four dilutions;
6. Protozoa identification by PCR.

The pregnant women and their NBs were submitted to peripheral blood sampling, from 3 mL to 5 mL, for laboratory analysis, between the 5th and 10th days of the NBs’ lives. The samples were kept refrigerated until the tests were processed in duplicate within a maximum period of 24 h after collection. The serology for the detection of anti-*T. gondii* IgG, IgM, and IgG avidity to confirm maternal infection and to assist in congenital toxoplasmosis diagnoses in newborns were performed at the Clinical Analysis laboratory of CH/UFG and the PCR tests in NBs were performed in the Laboratory of Biology, Physiology, and Immunology of Protozoa of Human Interest in the Tropical Pathology and Public Health Institute of UFG.

The sera from 88 puerperae and their respective NBs were analyzed, 48 of which were with anti-*T. gondii*-specific IgM AIP or reinfected puerperae during pregnancy, and 40 of which were CIP.

### 4.4. Laboratory Tests

#### 4.4.1. Detection of IgG and IgM Serum Levels and IgG Avidity Tests

The detection of anti-*T. gondii* IgG and IgM and the avidity tests were performed using the chemiluminescent technique (CMIA, chemiluminescent microparticle imunoassay), using Abbott Architect I4000 equipment (Chicago, IL, USA). These kits have sensitivity and specificity greater than 96%. The entire procedure was performed according to the manufacturer’s instructions. The concentrations of IgG, IgM, and IgG avidity of the samples were obtained using relative light units (RLU) with Abbott Architect I4000 equipment. The cut-off values considered for this study were equal to, or greater than, 300 IU/mL for IgG, >0.600 IU/mL for IgM, and for avidity testing, the IgG values <50.00% were considered low avidity, between 50.00–59.90% moderate avidity, and >60.00% high avidity.

#### 4.4.2. Fundoscopy

The fundoscopy for the evaluation of eye fundus involvement in NBs suspected of congenital toxoplasmosis was performed in the Reference Center for Ophthalmology of the Federal University of Goiás (RCOFU).

#### 4.4.3. Cranial Ultrasound

All NBs suspected of having been born with congenital toxoplasmosis were submitted to cranial ultrasound to identify possible brain involvement.

#### 4.4.4. Cerebrospinal Fluid Analysis

The analysis of the cytology, biochemistry, and serology in cerebrospinal fluid for toxoplasmosis were performed using the indirect immunofluorescence method with specific anti-*T. gondii* IgG and IgM identification.

### 4.5. Statistical Analysis

The data obtained were computerized in Microsoft Excel (2012) (Microsoft Corporation, Redmond, EUA). Statistical analysis was conducted with GraphPad Prism version 5.04 and the SPSS statistical package version 21.0. The differences were considered statistically significant at *p* < 0.05.

## 5. Conclusions

Early diagnosis is important for allowing an immediate therapeutic intervention for congenital diseases, thereby helping in the reduction of serious sequelae of disease and rational use of medicines that may have undesirable adverse effects in NBs and infants. In our study, the use of avidity test may be relevant for the diagnosis of congenital toxoplasmosis, showing that this tool could be adopted in prenatal and postnatal programs to assist in reducing the prevalence of severe congenital manifestations of toxoplasmosis. Newborns exposed to *T. gondii* with LA IgG presented increased serum levels of specific IgM and IgG, exhibited more severe congenital toxoplasmosis symptoms and had increased risk of developing toxoplasmosis than NBs exposed to *T. gondii* with HA, data not previously described in the literature. Despite present findings, studies with larger samples are needed to confirm these findings, we caution the importance of the monitoring of NB with LA for the early detection of congenital toxoplasmosis and appropriate treatment.

## Figures and Tables

**Table 1:** Comparative analysis between the IgG avidity test in newborns and their respective puerperae with acute and chronic toxoplasmosis in Goiania, Goias, Brazil, 2016.

	Newborn			Puerperae		
	LA	HA	GZ	LA	HA	GZ
**NB from AIP with LA**	100% (*n* = 17)	0% (*n* = 0)	0% (*n* = 0)	88% (*n* = 15)	0% (*n* = 0)	22% (*n* = 2)
**NB from AIP with HA**	0% (*n* = 0)	96.7% (*n* = 30)	3.33% (*n* = 1)	0% (*n* = 0)	100% (*n* = 31)	0% (*n* = 0)
**NB from CIP**	0% (*n* = 0)	100% (*n* = 40)	0% (*n* = 0)	0% (*n* = 0)	100% (*n* = 40)	0% (*n* = 0)
**Total**	19.32% (*n* = 17)	79.54% (*n* = 70)	1.14% (*n* = 1)	17.94% (*n* = 15)	80.68% (*n* = 71)	2.27% (*n* = 2)

NB: newborn; AIP: acutely-infected puerperae; CIP: chronically-infected puerperae; IgG avidity test values obtained using the chemiluminescent technique: LA: low avidity; HA: high avidity GZ: gray zone: (avidity values between 50.00–59.90%); Fisher’s test *p* < 0.0001, considering *p* < 0.005 as a statistically significant difference.

**Table 2:** Correlation degree between specific anti-*Toxoplasma gondii* IgG and IgM serum levels and IgG avidity tests in blood samples of newborns and puerperae infected by *Toxoplasma gondii*, in Goiania, Goias, Brazil, 2016.

		Puerperae IgG	NB IgM	Puerperae IgM	IgG Avidity in NB	IgG Avidity in Puerperae
**NB IgG**	R	0.809	0.460	0.179	–0.170	–0.450
	*p*	<0.001 *	0.001 *	0.224	0.113	<0.001 *
**Puerperae IgG**	R		0.274	0.177	–0.199	–0.535
	*p*		0.059	0.230	0.064	<0.001 *
**NB IgM**	R			0.835	–0.559	–0.535
	*p*			<0.001 *	<0.001	<0.001 *
**Puerperae IgM**	R				–0.568	–0.574
	*p*				<0.001 *	<0.001 *
**IgG Avidity in NB**	R					0.256
	*p*					0.016 *

The Pearson correlation coefficient (R) varies from –1 to 1. The signal indicates positive (directly proportional) or negative (inversely proportional) direction of the relationship between the variables and the value, that is, this coefficient measures the strength of the relationship. R values from 0.10 to 0.30 have a weak relationship; *R* = 0.40 to 0.6 represents a moderate relationship, and *R* = 0.70 to 1 indicates a strong relationship [43]. * indicates a statistically significant difference (*p* < 0.005).

**Table 3:** Distribution of clinical signs compatible with congenital toxoplasmosis in newborns infected by *Toxoplasma gondii* based on IgG avidity test values obtained using the chemiluminescent technique in Goiania, Goias, Brazil, 2016.

Clinical Manifestations	NB with Low Avidity of IgG	NB with High Avidity of IgG	*p* Value
Absence	43.8% (7/16)	68.7% (11/16)	0.0006 *
Presence	56.2% (9/16)	31.3% (5/16)	
Cortical-subcortical Dysfunction	11.1% (1/9)	–	
Cerebral Calcification	22.2% (2/9)	80.0% (4/5)	
Prematurity	11.1% (1/9)	–	
Hydrocephaly	44.4% (4/9)	–	
Systemic Toxoplasmosis	11.1% (1/9)	–	
Microcephaly	11.1% (1/9)	–	
Hepatoesplenomegaly	22.2% (2/9)	–	
Blindness	22.2% (2/9)	–	
Generalized Lymph Node Form	–	20.0% (1/5)	
Neuro-optic Form	22.2% (2/9)	–	

Clinical signs of newborns infected with congenital toxoplasmosis (*n* = 32) were evaluated: 16 had LA and 16 had HA. Of LA-infected NBs, nine presented more than one clinical sign of the disease. Of HA-infected NBs, one presented more than one clinical sign of congenital infection. Test: Fisher’s exact test. * indicates a statistically significant difference (*p* < 0.005) between the proportion of NBs with the presence and absence of symptoms of congenital toxoplasmosis, in groups with low and high avidity.
